# Establishment and Characterization of NCC-MFS5-C1: A Novel Patient-Derived Cell Line of Myxofibrosarcoma

**DOI:** 10.3390/cells11020207

**Published:** 2022-01-08

**Authors:** Ryuto Tsuchiya, Yuki Yoshimatsu, Rei Noguchi, Yooksil Sin, Takuya Ono, Taro Akiyama, Jun Sugaya, Eisuke Kobayashi, Naoki Kojima, Akihiko Yoshida, Seiji Ohtori, Akira Kawai, Tadashi Kondo

**Affiliations:** 1 Division of Rare Cancer Research, National Cancer Center Research Institute, 5-1-1 Tsukiji, Chuo-ku, Tokyo 104-0045, Japan; rytsuchi@ncc.go.jp (R.T.); yyoshima@ncc.go.jp (Y.Y.); renoguch@ncc.go.jp (R.N.); ishin@ncc.go.jp (Y.S.); takuono@ncc.go.jp (T.O.); taakiyam@ncc.go.jp (T.A.); 2 Department of Orthopaedic Surgery, Graduate School of Medicine, Chiba University, 1-8-1 Inohana, Chuo-ku, Chiba 260-8670, Japan; sohtori@faculty.chiba-u.jp; 3 Department of Musculoskeletal Oncology, National Cancer Center Hospital, 5-1-1 Tsukiji, Chuo-ku, Tokyo 104-0045, Japan; jsugaya@east.ncc.go.jp (J.S.); ekobayas@ncc.go.jp (E.K.); akawai@ncc.go.jp (A.K.); 4 Department of Diagnostic Pathology, National Cancer Center Hospital, 5-1-1 Tsukiji, Chuo-ku, Tokyo 104-0045, Japan; naokojim@ncc.go.jp (N.K.); akyoshid@ncc.go.jp (A.Y.)

**Keywords:** sarcoma, myxofibrosarcoma, patient-derived cell line, drug screening, proteasome inhibitor, histone deacetylase inhibitor

## Abstract

Myxofibrosarcoma (MFS) is a highly aggressive malignancy with complex karyotypes and a postoperative recurrence tendency, owing to its strong invasiveness. Although systemic chemotherapy is considered in patients with unresectable MFS, the efficacy of conventional chemotherapy is hitherto unclear. Recently, drug screening analysis using a large number of tumor cell lines has been attempted to discover novel therapeutic candidate drugs for common cancers. However, the number of MFS cell lines is extremely small because of its low incidence—this hinders the conduction of screening studies and slows down the development of therapeutic drugs. To overcome this problem, we established a novel MFS cell line, NCC-MFS5-C1, which was shown to harbor typical MFS genetic abnormalities and thus had useful properties for in vitro studies. We conducted the largest integrated screening analysis of 210 drugs using NCC-MFS5-C1 cells along with four MFS cell lines, which we previously reported. Bortezomib (a proteasome inhibitor) and romidepsin (a histone deacetylase inhibitor) showed stronger antitumor effects than the standard drug, doxorubicin. Therefore, the NCC-MFS5-C1 cell line can potentially contribute to elucidating MFS pathogenesis and developing a novel MFS treatment.

## 1. Introduction

Myxofibrosarcoma (MFS) is defined as a fibroblastic malignancy with variably myxoid stroma, pleomorphism, and a distinctive curvilinear vascular pattern [1]. MFS is one of the most common soft tissue sarcomas in elderly patients, although the incidence is extremely low (<0.1/100,000/years) [1,2]. The peak incidence occurs between the fifth and seventh decades of life, with a slight male predominance [3,4,5]. MFS most commonly occurs in the extremities, with a higher incidence in the lower than in the upper extremities [3,4,5,6]. Unlike other sarcomas, MFS often develops in superficial tissues [3,5]. Superficial MFS demonstrates strong infiltration through the fascia, with a characteristic finding called “tail sign” on magnetic resonance imaging (MRI) [7,8,9,10]. In contrast, deep-seated MFS often exists as a single discrete mass [6]. Genetically, MFS exhibits highly complex karyotypes associated with intratumoral heterogeneity and chromosome number aberrations [1,6]. Integrated genetic analysis has revealed that MFS often harbors mutations and copy number alterations (CNAs) of *TP53* (46%) and *RB1* (18%), as well as *CDKN2A* (16%) and *CDKN2B* (16%) [11]. In addition, the amplification of chromosome 5p, where multiple oncogenes such as *TRIO*, *RICTOR*, *SKP2*, and *AMACR* are located, is considered to be related to the malignant potential of MFS [12,13,14,15]. Clinically, the high MFS local recurrence rate poses a major problem. Due to its strong invasiveness, tumor resection is often incomplete, leading to local recurrence in 20–40% of cases [1,3,4,5,6]. Moreover, distant metastases occur in 20–35% of cases, resulting in a poor prognosis (5 year survival rate: 65–70%) [1,3,4,5,6]. Although chemotherapy and/or radiotherapy are used in unresectable MFS management, the clinical efficacy of these treatment modalities has not been well clarified, as no randomized trials have been conducted along these lines [6]. Therefore, novel therapeutic methods are needed for MFS treatment.

Patient-derived cell lines are fundamental tools for basic research. In particular, cell lines that have not undergone many passages retain the genetic characteristics of the corresponding original tumor and are useful for understanding the molecular biology of tumors [16,17,18]. In recent years, the concept of pharmacogenomics—a screening study to reveal the genetic characteristics and drug sensitivity of tumors using a large number of cell lines—has led to the identification of novel drugs for common cancers [19,20,21,22,23]. The rarity of cancers, including sarcomas, makes it difficult to conduct clinical trials, and the enormous cost of drug discovery has prevented the development of novel drugs [24,25]. Therefore, drug repurposing is an attractive way to identify novel candidate drugs for rare cancers [26]. However, there is a very small number of sarcoma cell lines [27]. To the best of our knowledge, only 12 MFS cell lines have been reported to date, and only two cell lines are publicly available (Table S1). Many cell lines are required to conduct a drug screening study; hence, cell lines of rare cancers, such as MFS, should be actively established. 

Thus, we established an MFS cell line and named it NCC-MFS5-C1 using surgical specimens of MFS. In addition to a detailed characterization of the cell line, we attempted to identify novel candidate drugs by integrating the results of drug screening studies for the following MFS cell lines, as reported in our previous studies: NCC-MFS1-C1 [28], NCC-MFS2-C1 [29], NCC-MFS3-C1 [30], and NCC-MFS4-C1 [31].

## 2. Materials and Methods

### 2.1. Patient History

**Figure 1:**
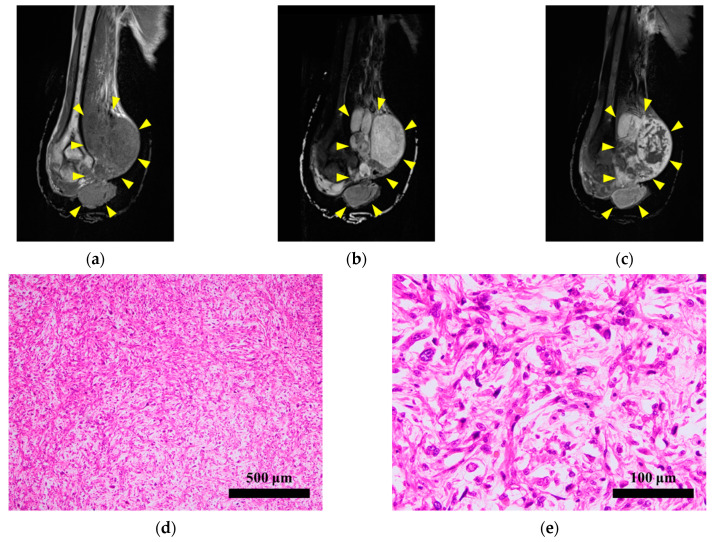
Clinical and pathological findings. Magnetic resonance imaging demonstrating a (**a**) low-intensity T1-weighted image and a (**b**) high-intensity STIR image of a soft tissue tumor around the right elbow, with a diameter of approximately 16 cm. (**c**) The periphery of the tumor was well enhanced by gadolinium. Yellow arrows indicate the tumor. (**d**,**e**) The tumor consisted of pleomorphic spindle cells with myxoid stroma and curvilinear vasculature.

The tumor specimen used in this study were derived from a 60-year-old man with MFS. The patient visited a hospital with a chief complaint of a mass in the right elbow. Needle biopsy indicated that the mass was malignant, and wide resection was recommended. However, the patient left the tumor untreated for 1 year. As a result, the tumor grew larger and was accompanied by bleeding. The patient visited the National Cancer Center Hospital (Tokyo, Japan), where a large tumor was detected around the right elbow using MRI (Figure 1a–c). There was no evidence of metastases, and therefore right upper extremity amputation was performed without neoadjuvant therapy. Histologically, the tumor exhibited pleomorphic spindle cell proliferation and consisted of a myxoid stroma with curvilinear vasculature. No clear line of differentiation was identified (Figure 1d,e). The tumor was diagnosed as MFS grade 3 on the basis of these findings. A part of the resected tumor was used for primary cell culturing. There was no tumor recurrence 1 year postoperatively, and the patient was under careful observation.

### 2.2. Pathological Assessment

The tumor specimens were formalin-fixed and paraffin-embedded to prepare for 4 μm thick sections. The deparaffinized sections were stained with hematoxylin and eosin (H&E) and validated by expert sarcoma pathologists.

### 2.3. Primary Cell Culture

Primary cell culture was performed as previously described [30]. The resected tumor specimen was immediately minced with scissors and cultured in DMEM/F12 (Gibco, Grand Island, NY, USA) with 10% heat-inactivated fetal bovine serum (FBS; Gibco), 100 μg/mL penicillin, and 100 μg/mL streptomycin (Nacalai Tesque, Kyoto, Japan) at 37 °C in a humidified atmosphere with 5% CO_2_. The cells growing out of the tumor were maintained for 1 year and passaged more than 35 times.

### 2.4. Authentication and Quality Control

Short tandem repeats (STRs) were examined at 10 loci using the GenePrint 10 system (Promega, Madison, WI, USA), as previously described [32]. Genomic DNAs of the established cell line and the original tumor were prepared for PCR using AllPrep DNA/RNA Mini kits (Qiagen, Hilden, Germany). The STRs of the obtained DNAs were amplified, and the PCR products were analyzed using a 3500xL Genetic Analyzer (Applied Biosystems, Foster City, CA, USA). The acquired data were analyzed using the GeneMapper software (Applied Biosystems). We examined the STR profiles using a cell line database, Cellosaurus [33].

*Mycoplasma* contamination was also evaluated using the e-Myco *Mycoplasma* PCR Detection Kit (Intron Biotechnology, Gyeonggi-do, Korea) according to the instructions. Extracted cellular DNA was used for PCR, and the DNA sequence that was unique to *Mycoplasma* was amplified. The PCR product was electrophoresed in the agarose gel, and the gel was stained with Midori Green Advance stain (Nippon Genetics, Tokyo, Japan). Finally, images were acquired using the Amersham Imager 600 system (GE Healthcare, Little Chalfont, Amersham, UK).

### 2.5. Single-Nucleotide Polymorphism Array Analysis

Single-nucleotide polymorphism (SNP) array analysis was conducted using the Infinium OmniExpressExome-8 v1.4 BeadChip (Illumina, San Diego, CA, USA) according to the manufacturer’s protocol and a previously reported procedure [30]. The genomic DNAs of the established cell line and the original tumor were amplified and hybridized onto array slides in an iScan system (Illumina). Log R ratios were calculated using the Genome Studio 2011.1 (Illumina) and analyzed using R (version 4.0.3, R Foundation for Statistical Computing, http://www.R-project.org, accessed on 28 October 2021) and DNAcopy package (version 1.64.0, Bioconductor, https://bioconductor.org/, accessed on 28 October 2021). Chromosome locations with copy numbers > 3 or <1 were considered as amplifications and losses, respectively. Genes that showed CNAs were annotated using biomaRt package (version 2.46.0, Bioconductor) and GRCh 37 assembly in Ensembl (https://asia.ensembl.org/index.html, accessed on 28 October 2021).

### 2.6. Cell Proliferation Assay

Cell proliferation was assessed as previously reported [30]. We seeded cells (2.5 × 10^4^) in 24-well culture plates on day 0. We assessed the number of cells based on the absorbance at 450 nm after treatment with Cell Counting Kit-8 (Dojindo Laboratories, Kumamoto, Japan) (*n* = 3). The growth curve was obtained on the basis of the absorbance measured every other day until day 4. The population doubling time was acquired on the basis of the growth curve.

### 2.7. Spheroid Formation Assay

Spheroid formation ability was assessed as previously reported [30]. Cells (1 × 10^5^) were seeded in 96-well round-bottom ultra-low attachment microplates (Corning, Inc., Corning, NY, USA) and incubated for 3 days. Spherical colonies fixed with iPGell (Genostaff, Tokyo, Japan) were prepared for paraffin sections, which were stained with H&E.

### 2.8. Invasion Assay by Real-Time Cell Analyzer

Invasive potential was assessed using a real-time cell analyzer (xCELLigence, Agilent, Santa Clara, CA, USA) along with NCC-MFS1-C1 [28], NCC-MFS2-C1 [29], NCC-MFS3-C1 [30], and NCC-MFS4-C1 [31], as previously reported [32]. We seeded cells (4 × 10^4^) suspended in a serum-free DMEM/F12 medium in the upper chamber coated with BD Matrigel matrix (BD Biosciences, Franklin Lakes, NJ, USA) (*n* = 2). DMEM/F12 supplemented with 10% FBS (Gibco), 100 μg/mL penicillin, and 100 μg/mL streptomycin (Nacalai Tesque) were added to the lower chamber. The migration of cells from the upper to the lower chamber was measured by electronic sensors every 15 min for 72 h.

### 2.9. Tumorigenesis Assessment in Nude Mice

Animal experiments were conducted as previously reported [30]. We followed the guidelines of the Institute for Laboratory Animal Research at the National Cancer Center Research Institute. We prepared cells (1 × 10^6^) suspended in a 100-μL 1:1 mixture of BD Matrigel matrix and D-PBS (-) (Nakalai Tesque, Inc., Kyoto, Japan) and injected subcutaneously into female BALB/c nude mice (CLEA Japan, Inc., Tokyo, Japan) (*n* = 4). After 2 months, the tumors were surgically removed and prepared for H&E staining.

### 2.10. Drug Screening Test

Drug screening tests were performed as previously reported [30]. In this experiment, the antitumor effects of 210 drugs, including Food and Drug Administration (FDA)-approved anticancer agents (Selleck Chemicals, Houston, TX, USA; Table S2), were assessed. The cells (1 × 10^4^) were seeded in a 384-well plate and incubated for 1 day (*n* = 2). On day 2, 10 µM of each drug was added. On day 5, cell proliferation was measured on the basis of the absorbance at 450 nm after treatment with the Cell Counting Kit-8. Cell viability was calculated by comparing with the DMSO control. The acquired data were analyzed using the results of NCC-MFS1-C1 [28], NCC-MFS2-C1 [29], NCC-MFS3-C1 [30], and NCC-MFS4-C1 [31], which we previously reported. Quantile normalization was performed using R (version 4.0.3, limma package version 3.46.0, Bioconductor), and unsupervised hierarchical clustering was performed using the gplots package (version 3.1.0, CRAN, https://cran.r-project.org, accessed on 28 October 2021).

The half-maximal inhibitory concentration (IC_50_) value was calculated for drugs that were hit in the preceding screening study and standard treatment drugs, as previously reported [30]. The drugs were added to the similarly seeded cells at 10 different concentrations from 0.1 to 100,000 nM. On the basis of the cell viabilities calculated in the same way, we identified IC_50_ values using GraphPad Prism 9.1.1 (GraphPad Software, San Diego, CA, USA).

## 3. Results

### 3.1. Authentication and Quality Control of the Established Cell Line

The NCC-MFS5-C1 cell line was authenticated by analyzing the STRs at 10 loci (Table 1, Figure S1). The STR match ratio between the cells and the corresponding tumor was 96.3% according to the Tanabe algorithm [34], meeting the 80% threshold [35]. Furthermore, we confirmed that the STR pattern did not match any other cell lines according to Cellosaurus [33]. Thus, the NCC-MFS5-C1 cell line is a novel cell line. 

We also confirmed that NCC-MFS5-C1 was not contaminated with *Mycoplasma* (data not shown).

### 3.2. Genetic Feature of the Cell Line

**Figure 2:**
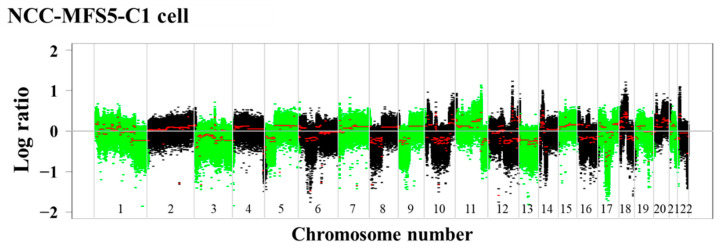
Single-nucleotide polymorphism array analysis. Focal copy number alterations were identified in NCC-MFS5-C1 cells. The *X*- and *Y*-axes indicate chromosomal location and the log ratio of copy number alterations, respectively.

Focal CNAs were identified in NCC-MFS5-C1 cells by SNP array analysis (amplification: 12q23.3; losses: 2q24, 5q14, 6q14, 7q21, 7p22, 8p23, 9p21, 12q15, 12q23.1, 16p13, and 17p13; Figure 2 and Table S3). Among the genes with losses, the tumor suppressor genes *CDKN2A* and *CDKN2B* in 9p21 and *TP53* in 17p13 were identified (Table 2).

### 3.3. Characterization of the Cell Line

**Figure 3:**
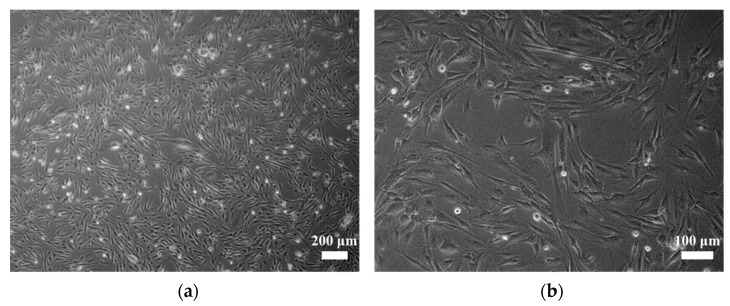

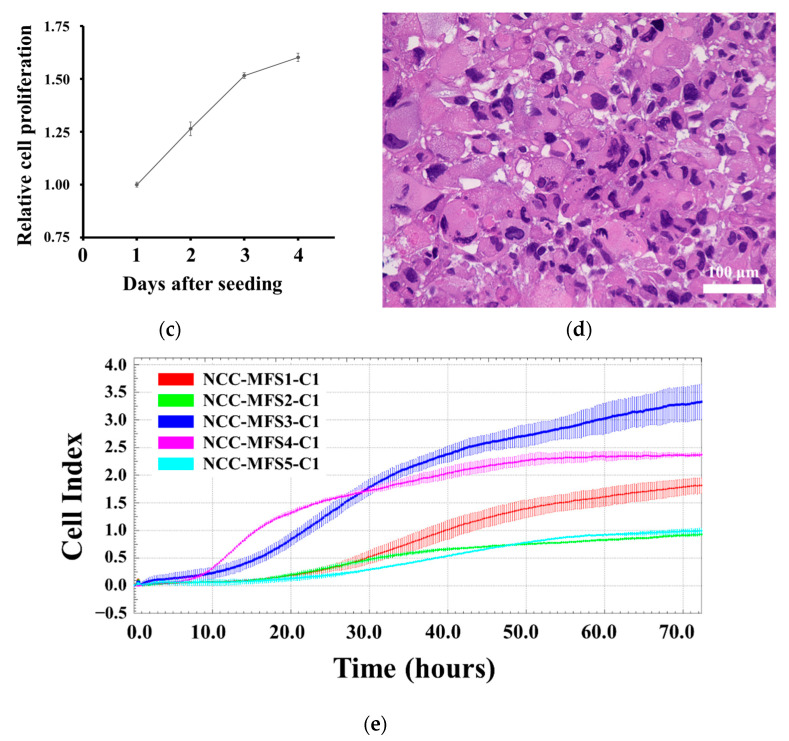
Characterization of NCC-MFS5-C1 cells. (**a**,**b**) NCC-MFS5-C1 cells exhibiting elongated spindle morphology under 2D culturing conditions. (**c**) Growth curve of NCC-MFS5-C1 cells, showing a constant cell growth. (**d**) H&E section of the spheroid showing pleomorphic oval cells with nuclear atypia. (**e**) The invasiveness of NCC-MFS5-C1 cells compared to that of MFS cell lines we previously established.

We characterized NCC-MFS5-C1 cells by assessing their morphology, growth ability, spheroid formation capability, and invasiveness. NCC-MFS5-C1 cells exhibited elongated spindle morphology under 2D culture conditions (Figure 3a,b). Moreover, the cells proliferated steadily, and the population doubling time based on the growth curve was 91.7 h (Figure 3c). NCC-MFS5-C1 cells had spheroid formation capability, and the H&E section of the spheroid showed pleomorphic oval cells with nuclear atypia (Figure 3d). The invasiveness of NCC-MFS5-C1 cells was comparable to that of NCC-MFS2-C1 cells, and inferior to that of NCC-MFS1-C1, NCC-MFS3-C1, and NCC-MFS4-C1 cells. The characteristics of MFS cell lines we established are summarized in Table S4.

### 3.4. Tumorigenesis in Nude Mice

NCC-MFS5-C1 cells were injected subcutaneously into nude mice that developed small tumors (Figure S2a), comprising spindle cells with an abundant myxoid matrix (Figure S2b,c). However, tumor size did not increase significantly.

### 3.5. Sensitivity to 210 Drugs

**Figure 4:**
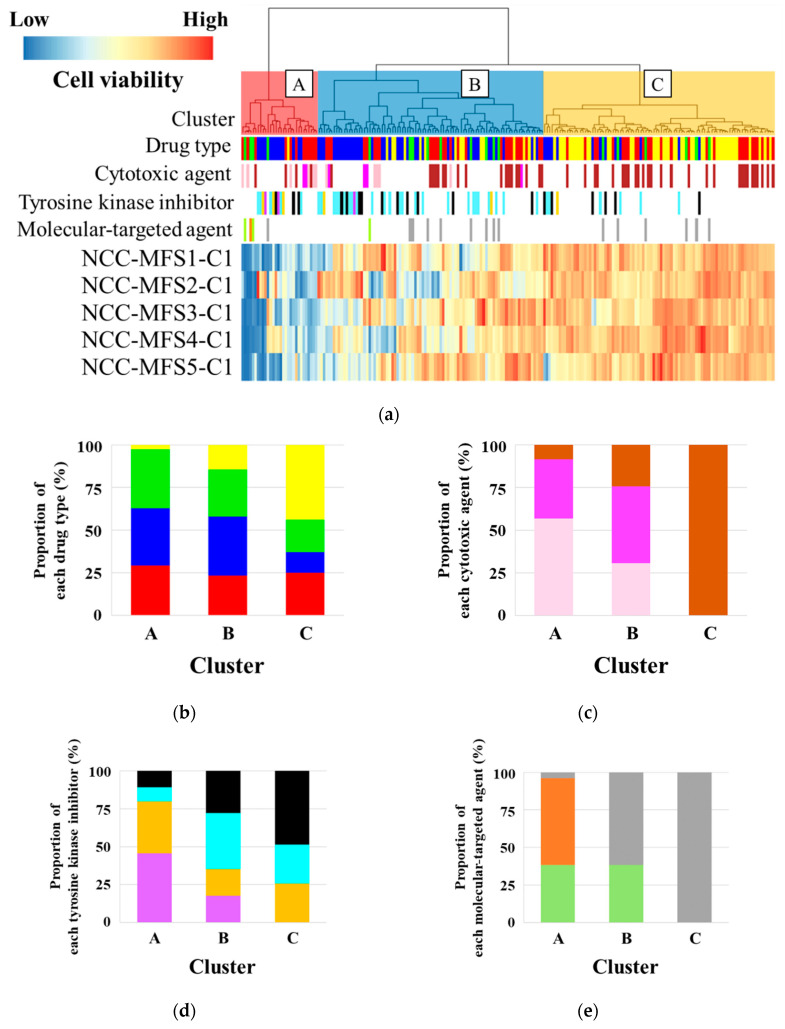
Drug screening tests on MFS cell lines. (**a**) The drugs were categorized into three groups according to their antitumor effect: cluster A, effective group; cluster B, intermediate effect group; cluster C, poor effect group. (**b**–**e**) The proportion of each drug type belonging to each cluster. The graphs are depicted after the normalization of the number of drugs. Data concerning NCC-MFS1-C1, NCC-MFS2-C1, NCC-MFS3-C1, and NCC-MFS4-C1 were previously reported [28,29,30,31].

We examined the antitumor effects of 210 drugs on NCC-MFS5-C1 cells. The cell viability of NCC-MFS5-C1 cells after drug treatment is summarized in Table S5, along with the cell viabilities of NCC-MFS1-C1 [28], NCC-MFS2-C1 [29], NCC-MFS3-C1 [30], and NCC-MFS4-C1 [31] cells, which we previously reported. The drugs were categorized into three groups according to their antitumor effect: cluster A, effective group; cluster B, intermediate effect group; cluster C, poor effect group (Figure 4a). The proportion of tyrosine kinase inhibitors and molecular-targeted agents was higher in clusters A and B than in cluster C (Figure 4b). Cluster A included a higher proportion of topoisomerase inhibitors than the other clusters (Figure 4c). As for tyrosine kinase inhibitors, ALK and BCR-ABL1 inhibitors occupied a high proportion in the effective group (Figure 4d). Among the molecular-targeted drugs, proteasome inhibitors belonged to only the effective group (Figure 4e).

**Figure 5:**
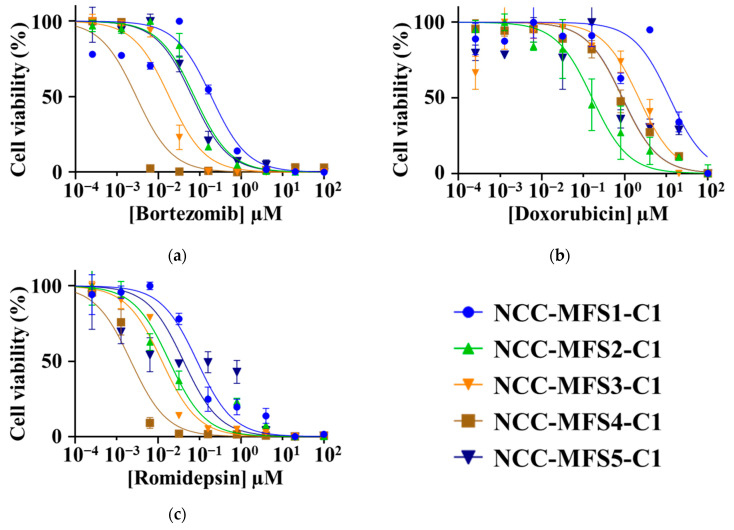
Cell viability of MFS cell lines at different concentration of each drug: (**a**) bortezomib, (**b**) doxorubicin, and (**c**) romidepsin. Data concerning NCC-MFS1-C1, NCC-MFSS2-C1, and NCC-MFS3-C1, and NCC-MFS4-C1 were previously reported [28,29,30,31].

The IC_50_ values of NCC-MFS5-C1 were calculated for the drugs that were hit in the screening study, and are summarized in Table S6, along with those of NCC-MFS1-C1 [28], NCC-MFS2-C1 [29], NCC-MFS3-C1 [30], and NCC-MFS4-C1 [31]. Notably, the proteasome inhibitor, bortezomib, and the histone deacetylase (HDAC) inhibitor, romidepsin, showed a more remarkable antitumor effect in all five MFS cell lines than doxorubicin, which is used as a standard drug for the treatment of sarcomas (Figure 5, Table 3).

## 4. Discussion

MFS is a highly aggressive malignancy with a poor prognosis. MFS often recurs due to its strong invasiveness, and the efficacies of conventional chemotherapy and radiotherapy have not been well clarified [6]. In recent years, drug screening studies have been conducted using a number of cell lines to identify novel candidate drugs for common cancers [19,20,21,22]. However, these drug screening studies have not been conducted in MFS due to the lack of MFS cell lines. In this study, we established a novel MFS cell line, NCC-MFS5-C1, from surgical specimens of MFS, and assessed its characteristics. We also performed a drug screening test; analyzed the results along with those of NCC-MFS1-C1 [28], NCC-MFS2-C1 [29], NCC-MFS3-C1 [30], and NCC-MFS4-C1 [31] cell lines; and successfully identified novel candidate drugs.

The NCC-MFS5-C1 cell line was established from a tumor specimen from an elderly male patient with MFS. Considering the epidemiology of MFS, the NCC-MFS5-C1 cell line was established from a typical MFS case. The original tumor occurred in the upper extremity, which is the second most frequent location of MFS [3,4,5,6]. The original tumor is often deep-seated without the “tail sign” on MRI [7,8,9]. Although MFS tends to occur in superficial tissues, deep-seated MFS is also often observed [3,5]. Therefore, NCC-MFS5-C1 may serve as a representative MFS cell line.

The NCC-MFS5-C1 cell line exhibited multiple CNAs, and was accompanied by chromosomal losses of *TP53*, *CDKN2A*, and *CDKN2B*, which are frequently observed in MFS. Among our previously reported MFS cell lines, NCC-MFS3-C1 [30] and NCC-MFS4-C1 [31] cell lines also harbored *CDKN2A* and *CDKN2B* losses. However, the loss of *TP53* was not observed in our previously reported MFS cell lines. Therefore, the NCC-MFS5-C1 cell line has different genetic features from the previously reported cell lines. CNAs of *CDKN2A* and *TP53* are frequently observed in histologically malignant MFS [15]. The NCC-MFS5-C1 cell line was established from a patient with histological grade 3 MFS and may reflect the malignant potential of the original tumor. Meanwhile, the NCC-MFS5-C1 cell line did not harbor the amplification of chromosome 5p, which is considered to be related to the malignant behavior of MFS [12,13,14,15]. MFS genetics have not been hitherto sufficiently investigated, and therefore further research is required.

The in vitro morphology of NCC-MFS5-C1 cells typified that of MFS cells. The NCC-MFS5-C1 cell spheroid formation capability may serve to elucidate the behavior of these cells under 3D conditions. Although NCC-MFS5-C1 cells had a malignant genetic potential, their population doubling time was slowest among the five cell lines that we established [28,29,30,31]. In addition, the invasive potential of the NCC-MFS5-C1 cell line was low compared to MFS cell lines we previously established. These findings suggest that other factors may not have been identified.

The drug screening test identified multiple promising drugs for the treatment of MFS. Remarkably, bortezomib (a proteasome inhibitor) and romidepsin (an HDAC inhibitor) demonstrated lower IC_50_ values than doxorubicin (a standard therapeutic drug for soft tissue sarcomas) in all five MFS cell lines; thus, bortezomib and romidepsin were considered to be promising candidate drugs. Bortezomib is an FDA-approved drug used for the treatment of multiple myeloma [36]. However, there is a paucity of studies focusing on the efficacy of proteasome inhibitors in MFS treatment. Only one study has examined the efficacy of bortezomib in MFS treatment [13]. In this study, *SKP2* overexpression was considered to be related to the malignant potential of MFS, and bortezomib showed its pharmacological effect through the inhibition of *SKP2*. In our study, bortezomib demonstrated antitumor effects in all five MFS cell lines; nevertheless, the NCC-MFS5-C1 cell lines and the other MFS cell lines that we previously established showed no amplification of the *SKP2* gene. Proteasome inhibitors, including bortezomib, may be promising candidate drugs for MFS treatment, albeit further studies are required to investigate the underlying pharmacological mechanisms. Romidepsin is approved for the treatment of cutaneous T-cell lymphoma. Few studies have focused on HDAC inhibition in MFS treatment. Although one study reported that the HDAC inhibitor OBP-801 demonstrated antitumor effects on two MFS cell lines, the underlying pharmacological mechanism remains unelucidated. A recent study revealed frequent amplifications of the *HDAC* gene family in soft tissue sarcomas [37]. This mechanism may account for the efficacy of romidepsin in MFS treatment.

This integrated drug screening analysis of five MFS cell lines is the largest analysis that has ever been conducted. However, our study has several limitations. First, MFS is an extremely heterogeneous malignancy. Since the NCC-MFS5-C1 cell line results from the monoclonal proliferation of the original tumor cell, it may not be representative of all MFS, although it was established from a typical MFS case. Second, although this is the largest drug screening analysis conducted for MFS, the number of cell lines used is still insufficient compared to those used for common cancers. For example, over 100 lung cancer cell lines were used in a previous drug screening study [23]. It is extremely difficult to establish such a large number of cell lines in rare cancers, such as MFS. However, we believe that it is important to continue the establishment of cell lines of rare tumors, such as MFS, and to actively share established cell lines among researchers to further elucidate the pathogenesis of MFS.

## 5. Conclusions

We successfully established a novel MFS cell line, NCC-MFS5-C1, from a typical case of MFS. NCC-MFS5-C1 cells exhibited multiple CNAs often observed in MFS cells and retained the genetic characteristics of MFS. The in vitro characteristics of NCC-MFS5-C1 cells may also serve as a basis for research on MFS. Drug screening analysis revealed that bortezomib (a proteasome inhibitor) and romidepsin (an HDAC inhibitor) showed a more prominent antitumor effect on MFS than the standard drug, doxorubicin. We believe that this novel MFS cell line contributes to the elucidation of MFS pathogenesis and the development of a novel MFS treatment.

## Figures and Tables

**Table 1:** Short tandem repeat analysis.

Microsatellite (Chromosome)	NCC-MFS5-C1	Original Tumor Tissue
Amelogenin (X Y)	X, Y	X, Y
TH01 (3)	8, 9	8, 9
D21S11 (21)	30, 32	30, 32
D5S818 (5)	11	11
D13S317 (13)	11	11
D7S820 (7)	11, 12	11, 12
D16S539 (16)	9	9, 11
CSF1PO (5)	11	11
vWA (12)	15, 18	15, 18
TPOX (2)	8	8

**Table 2:** Representative copy number alterations.

Gene Symbol	Chromosome Region	Copy Number	Type
CDKN2A	9p21.3	0.1	Loss
CDKN2B	9p21.3	0.1	Loss
TP53	17p13.1	0.1	Loss

**Table 3:** Half-maximal inhibitory concentration (IC_50_) values (µM).

Drug	NCC-MFS1-C1	NCC-MFS2-C1	NCC-MFS3-C1	NCC-MFS4-C1	NCC-MFS5-C1
Bortezomib	0.1895	0.07409	0.01825	0.002938	0.06552
Doxorubicin	12.00	0.1636	2.168	0.8678	0.8828
Romidepsin	0.08751	0.01877	0.01245	0.00222	0.04137

Data concerning NCC-MFS1-C1, NCC-MFS2-C1, NCC-MFS3-C1, and NCC-MFS4-C1 were previously reported [28,29,30,31].

## Supplementary Materials

The following are available online at https://www.mdpi.com/article/10.3390/cells11020207/s1, Figure S1: Short tandem repeat analysis. Figure S2: Tumorigenesis in nude mice. Figure S3: Growth curves for the IC_50_ value calculation. Table S1: List of myxofibrosarcoma cell lines. Table S2: List of 210 drugs examined in this study. Table S3: List of copy number alterations. Table S4: Summary of MFS cell lines we established. Table S5: Cell viability after treatment with 210 drugs (%). Table S6: IC_50_ values.
