# Supplementary figures and images for: Establishment and Characterization of NCC-MFS5-C1: A Novel Patient-Derived Cell Line of Myxofibrosarcoma
Source: Cells. 2022 Jan 8;11(2):207. doi: 10.3390/cells11020207 (PMC8773631; doi:10.3390/cells11020207)

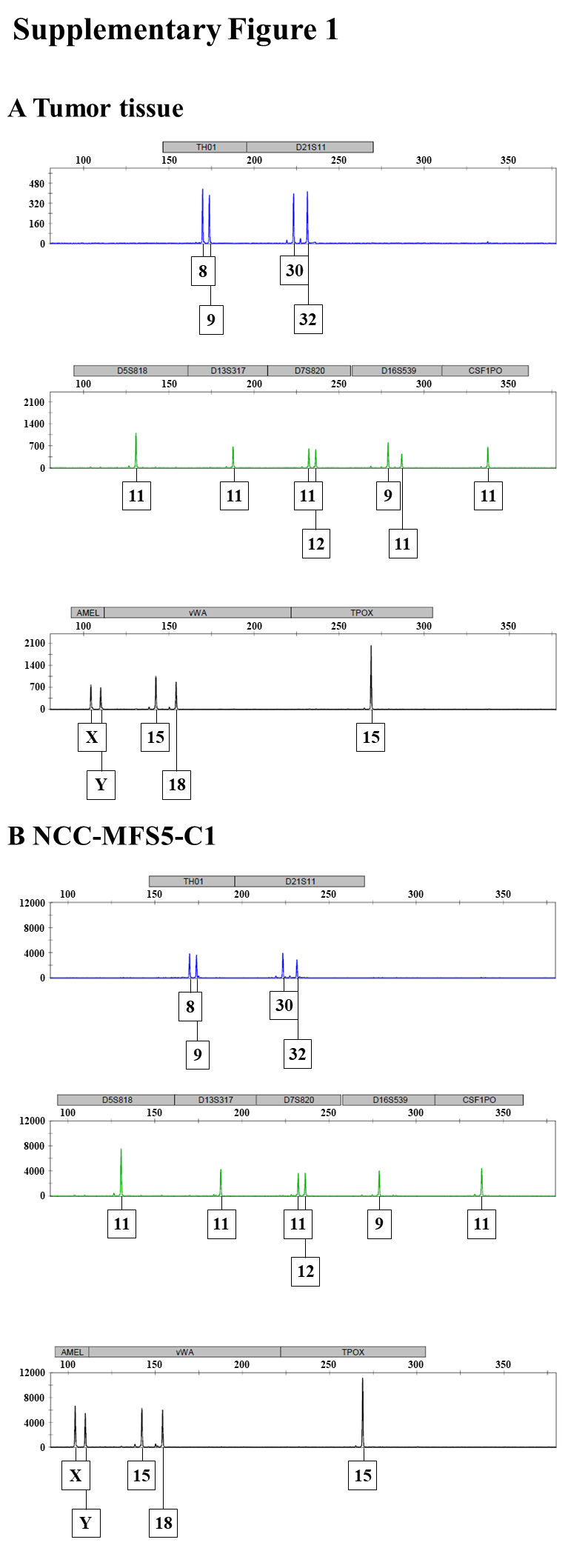

Supplement: Supplementary file 1 [file cells-11-00207-s001.zip › Supplementary Figure S1 STR.tif]

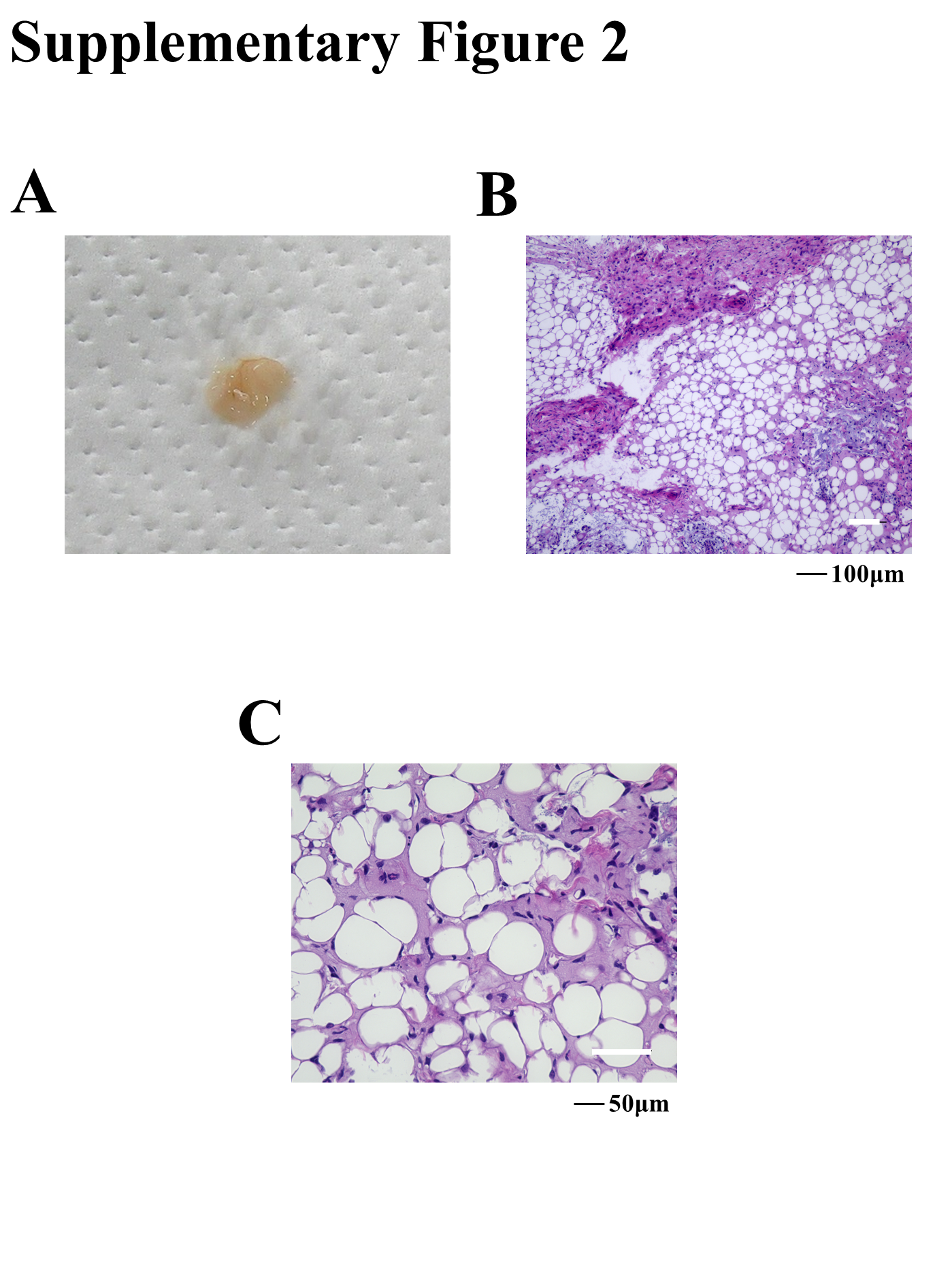

Supplement: Supplementary file 1 [file cells-11-00207-s001.zip › Supplementary Figure S2 animal experiment.tif]

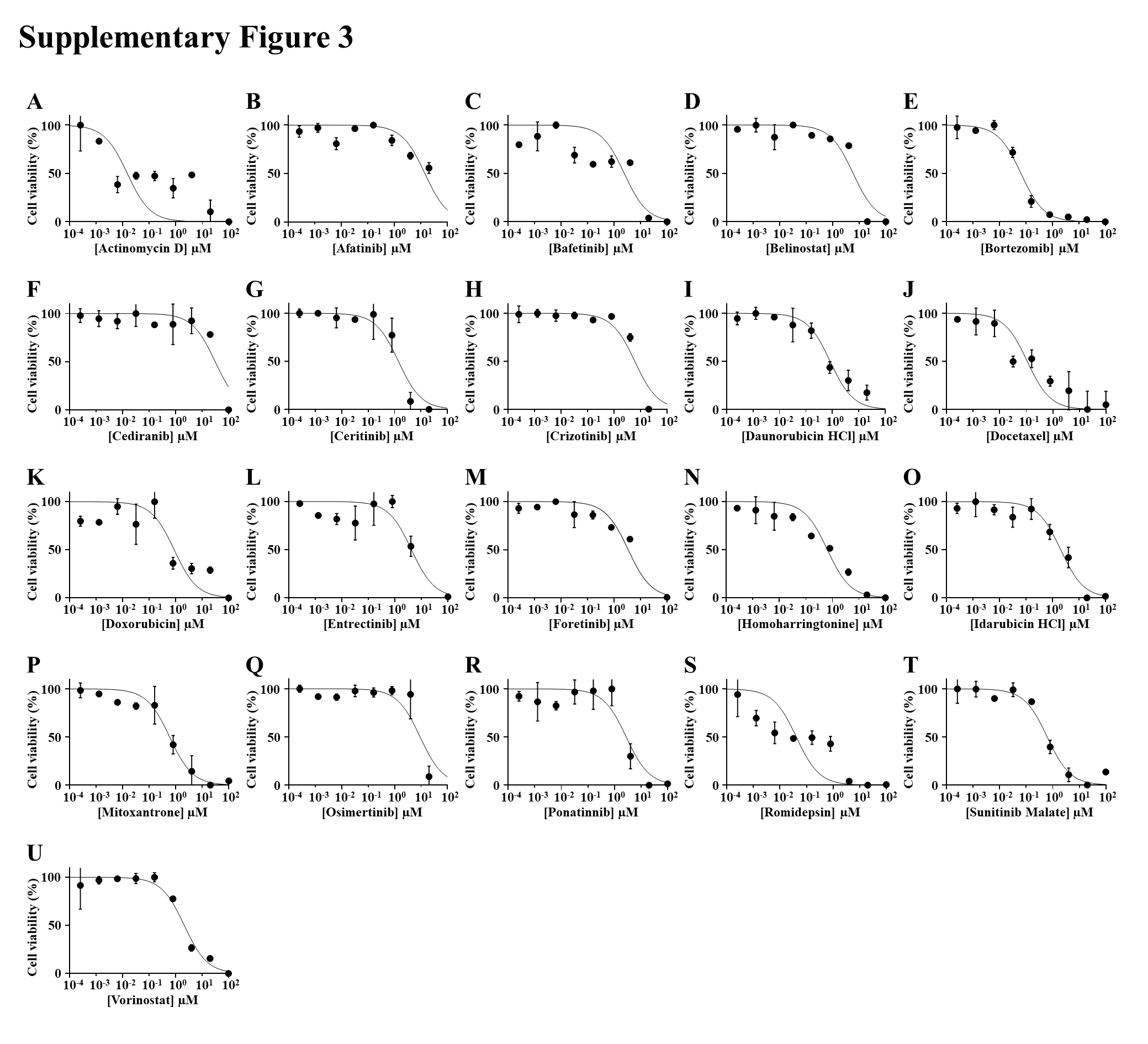

Supplement: Supplementary file 1 [file cells-11-00207-s001.zip › Supplementary Figure S3 IC50 of all.tif]
